# Hypertension Prevalence, Awareness, Treatment, and Control Among Adults Aged ≥18 Years — Los Angeles County, 1999–2006 and 2007–2014

**DOI:** 10.15585/mmwr.mm6632a3

**Published:** 2017-08-18

**Authors:** Craig M. Hales, Margaret D. Carroll, Paul A. Simon, Tony Kuo, Cynthia L. Ogden

**Affiliations:** ^1^Division of Health and Nutrition Examination Surveys, National Center for Health Statistics, CDC; ^2^Los Angeles County Department of Public Health.

Hypertension is an important and common risk factor for heart disease and stroke, two of the leading causes of death in adults in the United States. Despite considerable improvement in increasing the awareness, treatment, and control of hypertension, undiagnosed and uncontrolled hypertension remain public health challenges (*1*). Data from the National Health and Nutrition Examination Survey (NHANES) were used to estimate the prevalence of hypertension, as well as awareness, treatment, and control of hypertension among adults aged ≥18 years in Los Angeles County compared with adults aged ≥18 years in the United States during 1999–2006 and 2007–2014. During 2007–2014, the prevalence of hypertension was 23.1% among adults in Los Angeles County, lower than the prevalence of 29.6% among all U.S. adults. Among adults with hypertension in Los Angeles County, substantial improvements from 1999–2006 to 2007–2014 were found in hypertension awareness (increase from 73.8% to 84.6%), treatment (61.3% to 77.2%), and control (28.5% to 48.3%). Similar improvements were also seen among all U.S. adults. Although the prevalence of hypertension among adults in Los Angeles County meets the Healthy People 2020 (https://www.healthypeople.gov/) goal of ≤26.9%, continued progress is needed to meet the Healthy People 2020 goal of ≥61.2% for control of hypertension.

NHANES is a cross-sectional survey designed to monitor the health and nutritional status of the civilian noninstitutionalized U.S. population, and is conducted continuously in 2-year cycles. The NHANES sample is based on a complex, multistage probability design that includes oversampling of particular population subgroups to obtain reliable estimates for these groups. During 1999–2006, Mexican Americans, and during 2007–2014, all Hispanics (including Mexican Americans) were among the subgroups oversampled. Because of the size and population density of Los Angeles County and the large Mexican American/Hispanic population, Los Angeles County is a primary sampling unit that was selected with certainty in each 2-year NHANES cycle and weights were calculated to match the population totals for Los Angeles County (*2*,*3*). Data were aggregated over 1999–2006 and 2007–2014 to provide adequate sample size for Los Angeles County. All prevalences were estimated using the examined sample, for which the overall NHANES response rate was 77.3% during 1999–2006 and 72.6% during 2007–2014.

NHANES includes interviews conducted in the participant's home and a standardized physical examination that includes measurement of blood pressure conducted in a mobile examination center.* Hypertension is defined as a mean systolic blood pressure of ≥140 mmHg, a mean diastolic blood pressure of ≥90 mmHg, or current use of medication to lower blood pressure (*4*,*5*). Awareness of and treatment for hypertension were self-reported.^†^ Controlled hypertension was defined as having a mean systolic blood pressure <140 mmHg and a mean diastolic blood pressure <90 mmHg among persons with hypertension (*4*,*5*). Pregnant females were excluded from analyses (*4*). The Los Angeles County study sample included 975 adults during 1999–2006 and 1,084 adults during 2007–2014, and the U.S. sample included 19,989 adults during 1999–2006 and 23,647 adults during 2007–2014.

For all estimates, examination sample weights were used; analyses were performed using statistical software to account for the complex sample design. All reported prevalence estimates for adults aged ≥18 years were age-adjusted based on the 2000 U.S. Census projected population (*6*). All reported estimates of awareness, treatment, and control of hypertension for adults aged ≥18 years were age-adjusted using the subpopulation of persons who have hypertension in NHANES 2007–2008 (*4*). Standard errors of prevalences were estimated using Taylor series linearization and 95% confidence intervals were constructed using Korn and Graubard’s method for use with small expected positive counts (*7*). Differences in prevalence of hypertension, awareness, treatment, and control by sex, age group, race, and Hispanic origin, and between the U.S. and Los Angeles County were evaluated by examining p-values calculated using a univariate two-sided t-statistic, with the combined standard error accounting for the correlation between Los Angeles County and the United States (*8*). All differences reported are statistically significant (p<0.05). No adjustments were made for multiple comparisons. Estimates with a relative standard error >30% were designated as potentially unreliable and should be interpreted with caution. Population counts were calculated using the civilian noninstitutionalized population of Los Angeles County from the 2008–2012 5-year American Community Survey.

During 1999–2006, the age-adjusted prevalence of hypertension among adults was similar in Los Angeles County (28.0%) and the United States (29.6%); however, during 2007–2014, the age-adjusted prevalence of hypertension among adults was lower in Los Angeles County (23.1%, 1.7 million adults), compared with the United States (29.6%). Among adults with hypertension in Los Angeles County, from 1999–2006 to 2007–2014, awareness increased from 73.8% to 84.6%, treatment increased from 61.3% to 77.2%, and control of hypertension increased from 28.5% to 48.3%. During 2007–2014, in Los Angeles County, approximately 300,000 adults were unaware of their hypertension, approximately 400,000 were not being treated for hypertension, and approximately 800,000 did not have their hypertension controlled. Levels of awareness, treatment, and control of hypertension were similar in Los Angeles County and the United States during both 1999–2006 and 2007–2014 (Figure).

**FIGURE:**
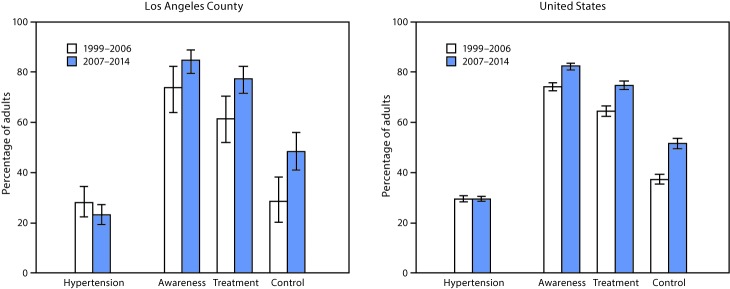
Age-adjusted prevalence* of hypertension,^†^ and awareness, treatment, and control of hypertension^§^ among adults aged ≥18 years — Los Angeles County and United States, 1999–2006 and 2007–2014 **Source:** National Center for Health Statistics, CDC: National Health and Nutrition Examination Survey (NHANES). * Hypertension prevalence estimates were age-adjusted by the direct method to the 2000 U.S. Census population using the age groups 18–39, 40–59, and ≥60 years. Estimates for awareness, treatment, and control of hypertension were age-adjusted using the subpopulation of persons who have hypertension (age groups 18–39, 40–59, and ≥60 years) in NHANES 2007–2008. ^†^ Statistically significant (p<0.05) difference in prevalence of hypertension between Los Angeles County and the United States in 2007–2014. ^§^ Statistically significant (p<0.05) differences in awareness, treatment, and control of hypertension from 1999–2006 to 2007–2014 in both Los Angeles County and the United States.

In both Los Angeles County and the United States, the prevalence of hypertension among adults increased with age, but younger age groups in Los Angeles County had significantly lower prevalences of hypertension compared with their U.S. counterparts (3.0% compared with 7.5% and 22.5% compared with 32.5% in persons aged 18–39 years and 40–59 years, respectively) (Table). In Los Angeles County and the United States, non-Hispanic black adults had a significantly higher prevalence of hypertension compared with both Mexican American adults and non-Hispanic white adults. Non-Hispanic white adults and Mexican American adults in Los Angeles County had lower prevalences of hypertension than their counterparts in the United States (Table).

**TABLE:** Age-adjusted prevalence of hypertension, and awareness, treatment, and control of hypertension* among adults aged ≥18 years, by sex, age, or race and Hispanic origin — Los Angeles County and United States, 2007–2014

Characteristic	% (95% CI)								
	Total	Sex		Age group (yrs)			Race and Hispanic origin		
		Male	Female	18–39	40–59	≥60	White, non-Hispanic	Black, non-Hispanic	Mexican American
**Hypertension**									
LA County	**23.1 (19.1–27.3)^†^**	20.9 (17.0–25.3)^†^	24.7 (19.0–31.1)	3.0 (1.4– 5.6)^ †,§^	22.6 (16.5–29.8)^ †,¶^	61.6 (52.2–70.5)^ ¶,^**	17.3 (11.1–25.1)^†^	40.5 (30.6–50.9)^††^	22.9 (18.4–27.9)^ †.§§^
United States	**29.6 (28.7–30.5)**	30.3 (29.2–31.5)	28.7 (27.6–29.9)^¶¶^	7.5 (6.8– 8.2)	32.5 (30.9–34.1)^ ¶^	66.8 (64.9–68.5)^ ¶,^**	28.6 (27.5–29.8)	41.4 (39.7–43.0)^ ††^	26.8 (25.0–28.7)^ ††,§§^
**Awareness**									
LA County	**84.6 (79.5–88.8)**	81.4 (72.3–88.6)	87.8 (82.2–92.2)	—***	90.3 (81.7–95.7)^†^	87.8 (79.2–93.7)	91.1 (60.9–99.7)	86.6 (72.5–95.1)	84.7 (75.7–91.4)
United States	**82.6 (81.2–83.9)**	80.0 (78.2–81.7)	85.6 (84.1–87.1)^ ¶¶^	66.0 (60.2–71.4)	82.5 (79.9–84.8)^ ¶^	85.9 (84.4–87.3)^ ¶,^**	82.9 (81.0–84.7)	85.9 (84.1–87.6)^ ††^	76.9 (74.0–79.7)^ ††,§§^
**Treatment**									
LA County	**77.2 (71.4–82.3)**	71.8 (62.2–80.1)	83.3 (77.1–88.4)^ ¶¶^	—***	81.0 (68.9–90.0)	83.8 (74.8–90.6)	87.0 (59.0–98.6)	77.5 (60.3–89.8)	70.6 (58.0–81.3)
United States	**75.0 (73.2–76.7)**	71.1 (69.1–73.0)	79.5 (77.5–81.4)^ ¶¶^	48.6 (43.3–54.0)	72.9 (69.7–75.8)^ ¶^	81.7 (80.2–83.2)^ ¶,^**	75.8 (73.5–78.0)	77.7 (75.3–79.8)	68.9 (65.4–72.3)^ ††,§§^
**Control**									
LA County	**48.3 (40.9–55.8)**	47.0 (38.1–56.0)	48.0 (36.8–59.4)	—***	56.2 (43.2–68.6)	48.6 (39.0–58.2)	48.1 (18.3–79.0)	56.3 (37.2–74.2)	47.2 (34.3–60.4)
United States	**51.8 (49.6–53.9)**	49.3 (46.8–51.8)	55.2 (52.8–57.6)^ ¶¶^	37.2 (31.9–42.6)	55.0 (51.4–58.6)^ ¶^	52.0 (49.9–54.2)^ ¶^	54.5 (51.8–57.3)	47.2 (44.6–49.8)^ ††^	42.7 (38.7–46.7)^ ††,§§^

**Source:** National Center for Health Statistics, CDC: National Health and Nutrition Examination Survey (NHANES).

**Abbreviations:** CI = confidence interval; LA = Los Angeles.

* Hypertension prevalence estimates were age-adjusted by the direct method to the 2000 U.S. Census population using the age groups 18–39, 40–59, and ≥60 years. Estimates for awareness, treatment, and control of hypertension were age-adjusted using the subpopulation of persons who have hypertension (age groups 18–39, 40–59, and ≥60 years) in NHANES 2007–2008.

^†^ Significantly different from the United States.

^§^ Estimate might be unreliable because relative standard error >30%.

^¶^ Significantly different from age group 18–39 years.

** Significantly different from age group 40–59 years.

^††^ Significantly different from non-Hispanic white adults.

^§§^ Significantly different from non-Hispanic black adults.

^¶¶^ Significantly different from men.

*** Statistical reliability criteria not met because sample size (n = 17) was less than the required minimum.

During 2007–2014, a higher percentage of adults aged 40–59 years with hypertension in Los Angeles County were aware of their hypertension (90.3%) than were adults of the same age in the United States (82.5%), whereas levels of awareness were similar among adults aged ≥60 years in Los Angeles County (87.8%) and the United States (85.9%). In Los Angeles County, 84.7%, 86.6%, and 91.1% of Mexican American, non-Hispanic black, and non-Hispanic white adults with hypertension, respectively, were aware of their hypertension, but these differences were not statistically significant.

Among adults with hypertension during 2007–2014, a higher percentage of women than men reported taking antihypertensive medication in Los Angeles County (83.3% versus 71.8%), but hypertension control was similar in women (48.0%) and men (47.0%). In Los Angeles County, treatment and control of hypertension were similar in adults aged 40–59 years and ≥60 years.

In Los Angeles County during 2007–2014, treatment and control of hypertension among non-Hispanic white adults were 87.0% and 48.1%, respectively, 70.6% and 47.2% among Mexican American adults, and 77.5% and 56.3% among non-Hispanic black adults. However, the observed differences in treatment and control of hypertension by race and Hispanic origin were not statistically significant.

## Discussion

Los Angeles County has been included in every 2-year NHANES cycle; therefore, the prevalence of many health conditions can be estimated and compared with those in the U.S. population. The examination component of NHANES allows estimation of the prevalence of both diagnosed and undiagnosed hypertension, as well as awareness, treatment, and control of hypertension. During 2007–2014 the age-adjusted prevalence of hypertension among adults was significantly lower in Los Angeles County (23.1%) than in the United States (29.6%), and improvements were made in awareness, treatment, and control of hypertension from 1999–2006 to 2007–2014. However, during 2007–2014, a total of 1.7 million adults aged ≥18 years in Los Angeles County were estimated to have hypertension, including approximately 300,000 who were unaware of their hypertension, approximately 400,000 who were not being treated for hypertension, and approximately 800,000 whose hypertension was not controlled. Emerging federal, state, and local initiatives to identify and control undiagnosed or undertreated hypertension in the community currently focus on investments in team care, which include the use of nonphysician extenders such as community health workers, home self-measured blood pressure monitoring, and comprehensive medication management programs led by pharmacists.^§^ Recent measures have also included strategies to reduce excess sodium consumption, as recommended by the Million Hearts initiative (https://millionhearts.hhs.gov) (*9*).

The findings in this report are subject to at least two limitations. First, the smaller Los Angeles County sample size required aggregation over an 8-year time period to produce reliable estimates. Second, because of the low prevalence of hypertension in persons aged 18–39 years, awareness, treatment, and control of hypertension could not be estimated and statistical tests could not be performed for this age group in Los Angeles County. The smaller effective sample size also reduced the power to detect differences by age and race or Hispanic origin in Los Angeles County.

Although progress has been made in the diagnosis, treatment, and control of hypertension in Los Angeles County, the Healthy People 2020 goal for control of hypertension has not been met. NHANES will continue to be an important source of data for monitoring progress in hypertension prevalence, awareness, treatment, and control, as evidence-based practices, such as those promoted through the Million Hearts initiative, continue to be implemented in Los Angeles County (*10*).

**Summary**

**What is already known about this topic?**

Approximately one third of U.S. adults have hypertension, and only about half of these adults have their hypertension under control. Hypertension is an important and common risk factor for heart disease and stroke, two of the leading causes of death in adults.

**What is added by this report?**

The examination component of National Health and Nutrition Examination Survey (NHANES) allows estimation of the prevalence of both diagnosed and undiagnosed hypertension, as well as awareness, treatment, and control of hypertension in Los Angeles County. During 2007–2014 the age-adjusted prevalence of hypertension among adults was significantly lower in Los Angeles County (23.1%) than in the United States (29.6%). Among adults with hypertension in Los Angeles County, awareness, treatment, and control improved significantly from 1999–2006 to 2007–2014; however, more than half of these adults still did not have their hypertension under control.

**What are the implications for public health practice?**

NHANES will continue to be an important source of data for monitoring progress in hypertension prevalence, awareness, treatment, and control, as evidence-based practices, such as those promoted through the Million Hearts initiative, continue to be implemented in Los Angeles County.

## Footnotes

* NHANES examination methodology and analytic guidelines available at https://wwwn.cdc.gov/nchs/nhanes/Default.aspx.

† Awareness of hypertension was determined by an affirmative response to the question “Have you ever been told by a doctor or health professional that you had hypertension, also called high blood pressure?” Treatment for hypertension was defined based on affirmative responses to the following questions: “Because of your high blood pressure/hypertension, have you ever been told to take prescribed medicine?” and “Are you now following this advice to take prescribed medicine?”

§ CDC State and Local Public Health Actions to Prevent Obesity, Diabetes, and Heart Disease and Stroke: Fact Sheet and Community Profiles. http://healthyamericans.org/health-issues/wp-content/uploads/2016/09/1422.pdf.
